# Double plating via anterolateral and posterolateral approach for distal femoral fracture

**DOI:** 10.1016/j.tcr.2023.100803

**Published:** 2023-02-18

**Authors:** Yasuaki Yamakawa, Yasutaka Masada, Ryuichiro Okuda, Toshiyuki Matsumoto, Takenori Uehara, Masanori Yorimitsu, Tomoyuki Noda, Toshifumi Ozaki

**Affiliations:** a Department of Orthopedic Surgery, Kochi Health Sciences Center, Kochi, Japan; b Department of Orthopedic Surgery, Okayama University Hospital, Okayama, Japan; c Department of Orthopedic Surgery, Kawasaki Medical School General Medical Center, Okayama, Japan; d Department of Orthopedic Surgery, Kawasaki Medical School, Kurashiki, Okayama, Japan

**Keywords:** Double plate, Combined approach, Distal femoral fracture, Hoffa fracture

## Abstract

Although there are some reports highlighting the applicability of double plates in distal femoral fractures, there is no standard approach or fixation method for supracondylar fractures combined with posterior coronal shear fractures. We report a case of distal femoral fracture treated with a lateral locking plate and posterior buttress plate using anterolateral and posterolateral approaches from one incision.

A 70-year-old man was hit by a motorcycle and had an intra-articular distal femoral fracture involving a long medial proximal spike and a single lateral condyle fragment, with the lateral condyle fragment posteriorly displaced. A 12-cm lateral skin incision was made, and the joint was developed using a para-patellar approach from the anterior to iliotibial band. Posterior buttress plate fixation was successfully performed from behind the iliotibial band using a posterolateral approach, followed by cannulated cancellous screw and lateral locking plate fixation from the anterolateral window.

Combined anterolateral and posterolateral approaches from one incision enable intra-articular exposure and fixation based on fixation principles for lateral condyle fragments combined with supracondylar fracture.

## Introduction

Fixation of distal femoral fractures is generally involving the use of a single plate with the lateral or anterolateral approach; however, in cases of comminuted fractures, double-plate fixation may be used to improve fixation [1], [2], [3]. In the case of posterior coronal shear fractures, fixation with a buttress plate based on the AO basic fixation principle has also been performed. However, there is no standard approach or fixation method for posterolateral buttress plate-based fixation in distal femoral fracture cases. We report a case of distal femoral fracture treated with lateral locking plate and posterior buttress plate fixation using anterolateral and posterolateral approaches from one incision.

## Case

**Fig. 1:**
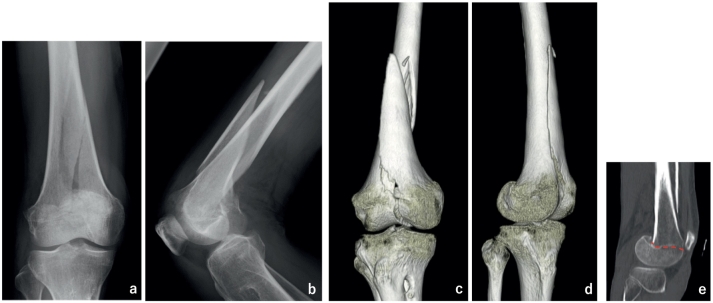
Plain X-ray and CT picture of the distal femur at the injury site. a: A-P plain radiograph, b: lateral plain radiograph, c:3D-CT showing intra-articular fracture of the distal femur, d: lateral condyle displaced posteriorly. e: Reconstructed CT coronal slice of the lateral condyle; the red dotted line shows the fracture line.

A 70-year-old man was hit by a motorcycle while walking. He had multiple brain contusions and a fracture of the right distal femur. It was a long spiral fracture extending from the joint to medial diaphysis, and the lateral condyle fragment was displaced posteriorly (Fig. 1a–e). He was diagnosed with AO/OTA classification 33C1.1. Five days after injury, surgery was performed in the supine position under general anesthesia.

### Surgery

**Fig. 2:**
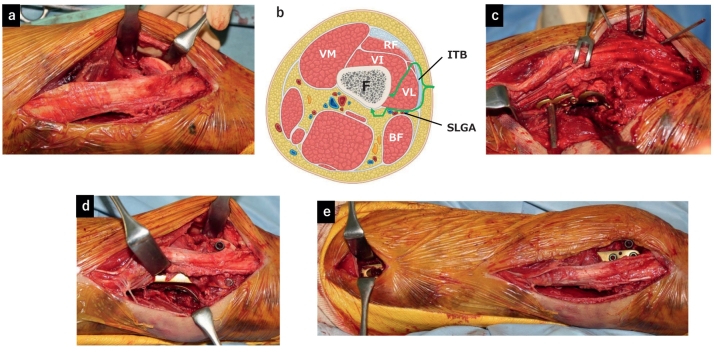
Intraoperative photograph and illustration of the surgical approach. a: Identification through the anterolateral window. b: Cross-sectional image of the distal femur. The long green arrows indicate the anterolateral and posterolateral windows. ITB: iliotibial band, SLGA: superior lateral genicular artery, VL: vastus lateralis muscle, RF: rectus femoris muscle and tendon, VM: vastus medialis muscle, VI: vastus intermedius muscle, BF: biceps femoris muscle, F: femur. c: Posterior buttress plate can be applied through the posterolateral window. d: Posterior part of the lateral condyle can be identified through the posterolateral window. e: LCP-DF plate was used as a minimally invasive technique.

**Fig. 3:**
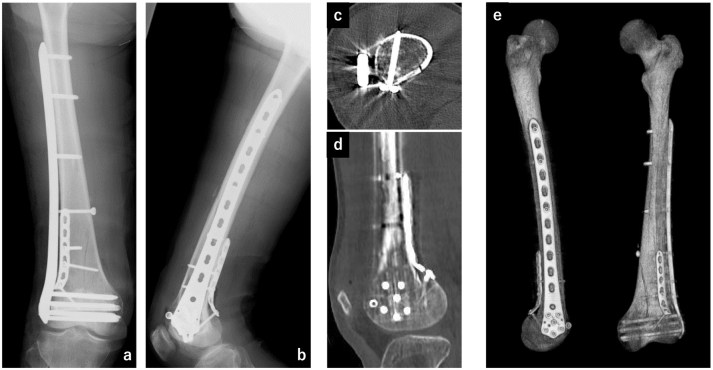
Postoperative plain X-ray and CT scan of the distal femur. a: A-P plain radiograph, b: lateral plain radiograph, c: CT axial slice image showing the perpendicular plate application. d: A buttress plate was applied at the ideal position of the posterior aspect of the distal femur lateral condyle. e: Postoperative 3DCT picture.

**Fig. 4:**
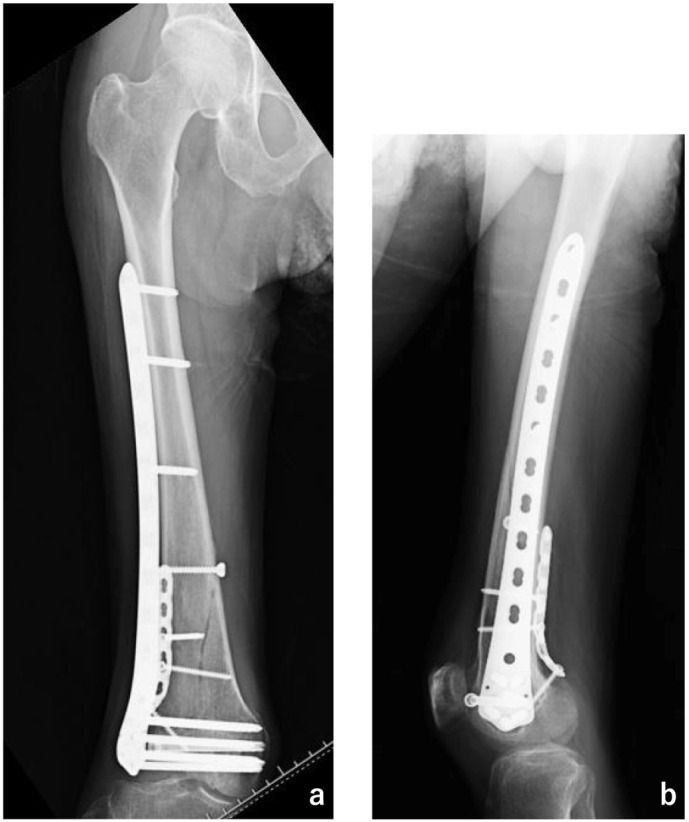
Plain X-ray at three month follow up. a: No displacement and bone union of the medial cortex was observed. b: Osteonecrosis of the lateral condyle is not evident.

A 12-cm skin incision was made proximally from Gerdy's tubercle through the lateral condyle of the femur. The anterior and posterior edges of the iliotibial band were identified and the joint surface was exposed from the anterolateral window using the lateral para-patellar approach (Fig. 2a). In addition, from the posterior edge of the iliotibial band, the vastus lateralis was avoided anteriorly and the biceps femoris posteriorly (Fig. 2b). A branch of the superior lateral genicular artery was then dissected. It is better not to dissect SLGA if possible. The lateral condyle and posterolateral areas of the metaphysis were exposed for the placement the plate and visualize the fracture line. For the reduction of the diaphysis, additional 3-cm incision was made anteromedial aspect of the femur and reduction was done with manual maneuver. After reduction of the articular surface, posterior condyle and diaphysis, temporally fixation with Kirshner wire was performed. Then the articular surface was fixed with a 6.5-mm cannulated cancellous screw and diaphysis was fixed with 4.5-mm cortical screw as a lag screw, respectively. A 3.5 metaphyseal plate (DePuy Synthes, Oberdorf, Switzerland) was bent and used as a buttress plate to fix the posterior lateral condyle (Fig. 2c). LCP-DF (DePuy Synthes, Oberdorf, Switzerland) was fixed as a neutralization plate with MIPO technique (Fig. 2d, e). Postoperative plain radiographs confirmed that anatomical reduction and fixation of the diaphysis and articular surfaces were achieved, and the two plates were perpendicular (Fig. 3a, b). Computed tomography confirmed anatomical reduction and good placement of the posterior buttress plate. Post-treatment was started without limiting the range of motion, but it was impossible to practice walking due to consciousness disturbance caused by brain contusion. Therefore, the patient was transferred to another hospital on the 10th day after injury. At the final 3 month follow-up, there was no evidence of fixation failure or osteonecrosis of the lateral condyle (Fig. 4), and walking was still impossible due to consciousness disturbance and range of motion of the knee was 0° of extension and 100° of flexion.

## Discussion

This case involved a distal femoral fracture that extended to the diaphysis combined with a posteriorly displaced lateral condyle fragment, although the lateral condyle was not a coronal plane fracture (Hoffa fracture). The specific mechanism of a Hoffa fracture is not well understood, but it is thought to occur when axial pressure is applied in a knee flexion position of 90° or more [4]. And also, it is reported that the greater the knee flexion angle at the time of injury, the greater the distance of the fracture line from the posterior cortex [4]. In this case, considering transvers fracture of the lateral condyle with the spike to the anteromedial side of the diaphysis, it is thought that the injury mechanism was due to combination force of deep flexion and varus of the knee. The frequency of supracondylar fractures of the femur with Hoffa fractures is reportedly 77 of 202 (38.1 %), of which 85 % of single condyle fractures were lateral condyle fractures [5]. Femoral supracondylar fractures combined with lateral Hoffa fractures are not thought to be rare.

For Hoffa fractures with large fragments (Letenneur classification types I and III), fixation with A-P screws or a posterior buttress plate is recommended [6]. However, this case had a transverse distal fracture line with posterior displacement, A-P lag screw was not suitable. And also, Sun et al. reported that in a biomechanical study of Letenneur type I Hoffa fractures, plate fixation resulted in stronger fixation than screw fixation alone [7]. It is also noted that a better fixation force can be obtained by placing the plate on the lateral side rather than on the posterior side. However, when using the lateral anatomical locking plate, it is difficult to use the lateral plate for Hoffa fracture fragments. Moreover, based on the AO basic fixation principle, the anti-glide plate to the posterior aspect was considered the best. Tetsunaga et al. reported good results with an LCP-DF and a twisted one-third tubular plate placed posterolateral side through a lateral approach for lateral Hoffa fractures [8], which we consider an alternative method. However, the lateral approach may result in insufficient posterior exposure, making fixation based on the AO principle difficult. Another study reported that lateral plate fixation was performed using a posterolateral approach [9], and intra-articular exposure was considered difficult. Another study reported that osteotomy of Gerdy's tubercle is useful for the extensive expansion of Hoffa fragments and reduction of the articular surface [10], the risk of osteotomy fragment nonunion remains.

The advantages of this method are direct exposure and reduction of the articular surface from the anterolateral approach, and the plate can be placed directly behind the lateral condyle fragment using the posterolateral approach. Furthermore, using a double plate, a strong mechanical fixation force can be obtained. However, there is a concern that dissection of the superior lateral genicular artery branch may cause blood flow disturbance to the posterior condyle [6]. There are vascular anastomoses around the knee including SLGA, and it is thought that blood flows from other blood vessels to the condyles. Although osteonecrosis of the condyle was not evident in this case, dissection of the SLGA in both at the parapatellar approach and the posterolateral approach may interrupt the blood flow to the condyles, so careful follow-up is necessary.

## Conclusion

Combined use of anterolateral and posterolateral approaches enables mechanically strong lateral and posterior double-plate fixation based on fixation principle for distal femoral fractures combined with lateral coronal plane fracture.

## Funding

None.

## Conflict of interest

None.
